# Efficacy of HyperDRYmist® technology in reducing residual environmental MDR bacterial contamination in tertiary hospital

**DOI:** 10.1186/2047-2994-4-S1-P41

**Published:** 2015-06-16

**Authors:** M Ferrari, A Bocconi, A Anesi, S Asticcioli, D Baroni, C Garzoni

**Affiliations:** 1 Department of Hygiene, AO Lodi, “Maggiore” Hospital of Lodi, Lodi, Italy; 2 Hospital Management, AO Lodi, “Maggiore” Hospital of Lodi, Lodi, Italy; 3 Department of Microbiology, AO Lodi, “Maggiore” Hospital of Lodi, Lodi, Italy; 4 Department of Internal Medicine and Infectious Diseases, Clinica Luganese, Lugano, Switzerland; 5 Department of Infectious Diseases, Inselspital; Bern University Hospital and University of Bern, Bern, Switzerland

## Introduction

Environmental persistence of multidrug-resistant (MDR) organisms in hospital environment is arduous to reduce or eliminate. Efficacy’s inconsistency characterizing manual disinfection that generates high levels of residual surface contamination compounds adversities in achieving reduction.

## Objectives

Study evaluated a novel no-touch disinfection technology named HyperDRYMist^®^, which decontaminates hard surfaces by delivering aerosolized enhanced Hydrogen Peroxide, as an addition to manual disinfection (terminal cleaning).

## Methods

Hard surfaces of 20 hospital rooms occupied by patients affected by classic MDR bacteria prospectively sampled for contamination in 10 standardized high-touch points following patient’s discharge. Measurements taken after manual disinfection with active chlorine (2000 ppm), before micro-nebulization via HDM^®^ and after it. Eventual residual bacterial contamination excluded by enriched broth culture.

## Results

For 8 months, prospectively identified and included 20 cases of “classic” MDR patients (see list below). After terminal cleaning, mean residual bacterial contamination was 59,27±78,89 (mean± SD) cfu/ml, with values reaching up to 400 cfu/ml, then reduced to 2,48±12,07 (p < 0.0001) after HDM^®^. Purposely searched for environmental presence of MDR after conventional cleaning. In 14/20 rooms MDRs bacteria still detected, in particular found MRSA in 4 of 4 rooms, Acinetobacter baumanii in 2/2, MDR Pseudomonas in 2/2, KPC in 2/4, VRE in 0/2, E.coli ESBL 2/2, K. pneumonia ESBL 0/2, Stenotrophomonas maltophilia 2/2. After HDM^®^ in none of the rooms MDR bacteria detected.

## Conclusion

Study confirms how significant levels of environmental contamination persist when solely manual disinfection is adopted to eradicate MDR bacteria from surfaces. In spite of quality certified protocols, overall inconsistency of results observed with degrees of efficacy clearly operator’s dependent. HDM^®^ brought consistency of results in drastically reducing pathogens’ presence.

Persistence of MDRs in rooms sampled suggests surfaces may play pivotal role for HAI transmission, hence the advantages of disinfection technologies like HDM^®^ in terminal cleaning procedures.

## Disclosure of interest

None declared.

